# Clinical Remarks on Dysentery

**Published:** 1855-08

**Authors:** 


### EDITORIAL.

*Clinical Remarks on Dysentery*, founded on cases treated in the Mercy Hospital, Chicago, and in private practice.

As the present is the season of the year when Dysentery is most prevalent, I have thought it not amiss to give a brief summary of my own clinical or bed-side experience during the past year. The whole number of cases of severe disease treated in the Mercy Hospital of this city, during the year 1854, was about 570; of which 42 were dysentery, 99 typhoid fever, 9 typhus, 49 diarrhœa, 30 cholera, 45 periodical fever, and 12 tubercular phthisis.

Nearly all the cases of dysentery were admitted in an advanced stage of the disease, and 18 had become chronic with evident ulceration of the bowels. Of the more recent cases, none were admitted between the first of January, 1854, and the first of July, and 24 between the last named date and the thirty-first day of December. If we divide the time more closely, we shall find that of the recent cases 23 were admitted during the months of Aug., Sept., and October; while of the chronic cases 14, or nearly four-fifths were admitted in the months of October and November. Of the 24 recent cases 18 recovered and 6 died. Of the 18 chronic cases 11 recovered and 7 died; making 13 deaths in 42 cases, or nearly one-third of the whole. Without explanation this would appear like a high ratio of mortality; but those classed as recent cases were all admitted in an advanced stage of the disease, and from among the poorer classes. Of the 6 deaths among them, two occurred during the first twenty-four hours after admission, one on the third day and two on the fifth. The average duration of the treatment in all the more recent cases was eight days; in those classed as chronic twenty-five days. To show the difference in results between hospital practice, where nearly all the acute diseases are brought in an advanced stage of their progress, and from among those classes who live in the most filthy streets and pay the least attention to hygienic regulations, and ordinary private practice in the same city, I will state that during the first 25 days of the present month (July 1855), I treated in private practice 49 cases of Dysentery, including adults and children, of which 48 recovered and 1 died. The duration of the treatment was from three to ten days. The character of the disease, as I have met with it during the present season, has been decidedly active or inflammatory. Most of the cases commence with a slight chilliness, followed by pains in the head, back and limbs; a flushed face ; dry, hot skin ; a white or yellowish white coat upon the tongue ; thirst ; restlessness ; a quick and firm pulse , very severe griping pains in the abdomen, with tenesmus ; and frequent discharges, at first of glairy mucus, plentifully mixed with blood, and subsequently of a whitish, flakey mucus, mixed with blood, but small in quantity. The urine is generally scanty and high colored, and in many cases accompanied by severe tenesmus, it becomes almost suppressed, or voided with much pain, and in very small quantities at a time. If the disease continues uncontrolled by treatment, the patient rapidly emaciates, and after six or eight days the features become pale and contracted, the skin remains dry but not hot, the tongue often presents a dry and and reddish-brown streak over the middle. The patient complains of less pain in the abdomen, but is still harrassed with the desire to go to stool often, sometimes sitting a long time and passing nothing, more frequently giving passage to a small quantity of muco-purulent matter, containing shreds of coagulated lymph and streaks of blood.

In other instances the discharge becomes more copious, consisting of serum rendered dark-red by the intermixture of blood, and holding suspended flakes of mucous or lymph. With such, as the disease advances, the eyes become deeply sunken, the lips often leaden color, the countenance haggard, the skin cool, the mind lethargic, and the pulse frequent and feeble. In a considerable number of cases the stomach is irritable from the commencement of the disease; and about the second or third day the vomiting becomes frequent, and the matter vomited consists of water colored yellow or slightly green, but subsequently it becomes a deep copper green, or sky blue color. In these cases where the treatment was neglected in the beginning, or badly managed, I have found this symptom exceedingly persistent and troublesome. It is almost always accompanied by a feeling of great oppression and distress at the epigastrium, which is somewhat relieved by each act of vomiting. In a few cases seen during the present summer, the distress at the epigastrium extended through the chest to the top of the sternum, accompanied by extreme anxiety, a sense of suffocation, and occasional palpitations of the heart. In nearly all of these bad cases, the symptoms have been distinctly *remittent*, they generally being the worst during the night, and ameliorated for two or three hours in the morning. On the other hand I have met with a few cases in which the constitutional or general symptoms were exceedingly slight, the patients complaining of little else than a frequent desire to defecate, with transient pains in the course of the colon, and bloody, mucous discharges.

TREATMENT.—In a disease so variable in its symptoms and degree of severity as dysentery, the treatment must of course vary also very much. In ordinary cases, when called during the first two or three days after the commencement of the disease, I direct for an adult something like the following, viz:

| | | |
|---|---|---|
| ℞ | Sub. murias. hydrarg., | 12 grs. |
| | Pulv. opii, | 10 grs. |
| | White sugar, | 30 grs. |

Mix and divide into six powders; one to be taken every two, three, or four hours, according to the severity of the symptoms, and continued until the discharges are stopped and the bowels have been quiet from eight to twelve hours at least. At the same time if the pain in the abdomen has been severe, I cover it with fomentations of hops, and keep up the action of the kidneys by giving between the powders a teaspoonful of the Spts. Nit. Dulcis. Generally after continuing this treatment 24 hours, I find the skin less hot and dry, the pulse less quick, the discharges either entirely suppressed or less frequent and painful, but the patient feeling dull and heavy. If the discharges from the bowels are entirely stopped, I allow them to remain so from 12 to 18 hours, and then move them by means of castor oil or seidleitz powders, being always careful to leave an anodyne sufficient to promptly quiet the bowels after two or three fecal evacuations have occurred. This treatment, which usually occupies about two days, is in some cases all that is required; but more frequently there remains some griping pains, with frequent disposition to go to stool, and discharges of a slimy character streaked with blood; but without much general febrile action. At this stage of the trouble I put the patients upon the use of the emulsion of oil of turpentine and laudanum, or of pills of nitrate of silver and opii, and sometimes of both, taken alternately. The formula for the emulsion has been several times published in the Journal in connexion with former reports, and is as follows, viz.:

| | | | |
|---|---|---|---|
| ℞ | Ol. Terebinth, | | ʒij. |
| | Tinct. Opii, | | ʒij. |
| | Pulv G. Arabac<br>White sugar, | aa. | ʒiij. |
| | Rub together thoroughly and add | | |
| | Mint Water, | | ℥ij. |

Mix, and give one teaspoonful every two or four hours, according to the frequency of the discharges. When I use the nitrate of silver, it is generally in the form of pills, as follows, viz.:

| | | |
|---|---|---|
| ℞ | Nitras Argenti, | 7 grs. |
| | Pulv Opii, | 20 grs. |

Mix and divide into 20 pills, of which one may be given every two, three or four hours. By these pills or the emulsion, or the two given alternately, at just such intervals as will suppress the pain and limit the discharges to from three to six in the twenty-four hours, the patients will generally entirely recover in from four to seven days.

While this is the case with a large majority of the cases I meet with, there are still many exceptions. For instance, in those cases described as connected with persistent vomiting, it is necessary to give the calomel combined with morphine, in divided doses often repeated, with strong sinapisms over the epigastrium, and repeated enemas of cold water and laudanum thrown into the rectum. And when the first febrile symptoms have abated, the skin become cool, the pulse soft and quick, and yet the patient restless with a sense of oppression at the lower end of the sternum, with occasional vomiting of green or blueish water, I have found the following mixture to succeed better than anything else in most cases, viz.:

| | | |
|---|---|---|
| ℞ | Chloride of Sodium, | ʒij. |
| | Tinct. Opii, | ʒij. |
| | Sulph Quinine, | 15 grs. |
| | Water, | ℥iv. |

Mix, and shake it well before using, and give a dessert spoonful every two or three hours.

At the same time give after each second evacuation an enema, consisting of half a teacupful of cold water, with one teaspoonful of laudanum and ten grs. of Quinine. Another preparation which I have found of much value, in cases where, after the general febrile symptoms have abated or entirely disappeared, the tenesmus, with disposition to be frequently on the stool and yet but little discharge, continues, is as follows, viz.:

| | | |
|---|---|---|
| ℞. | Strychnine, | 1 gr. |
| | Nitric Acid, | ʒj. |
| | Tinct. Opii, | ʒij. |
| | Water, | ℥ij. |

Mix, give one tea-spoonful every two or three hours in plenty of sweetened water.

In those few cases accompanied by decided *periodicity*, I add anti-periodic doses of the tannate of quinine to the other remedies indicated, with the most prompt benefit. In treating the dysentery in children, I use the same remedies as in the adult, only altering the dose to correspond with the age of the child. In them however, one of the most troublesome symptoms is a suppression of urine, especially while they are kept much under the influence of opiates. I have met with many cases of children under two years of age, to whom it was impossible to give sufficient opiates in any form, to quiet the bowels without so far suppressing the secretion from the kidneys, that the little sufferers become extremely restless and feverish, or so lethargic that they could scarcely be aroused at all. In such cases I have found the best effects from the following, viz.:

℞. Erigeron Canadensis, ℥ss
Lycopus Virginicus, ℥ss.
Boiling water 1 pint. Make an infusion, and when cold give to an adult one or two tablespoonsful every hour or two. To children under two years a teaspoonful may be given every half hour or hour. In cases where the patients are much reduced and the skin cool, the Tannate of Quinine may be added to the infusion with benefit.

During the last month, I met with two or three young children affected with severe dysentery, which resisted every means I could adopt for several days. Opiates, either with or without alteratives and astringents, invariably suppressed the urinary secretion, and increased the nausea and vomiting, or rendered the patients lethargic to a dangerous degree. In all these I found very much benefit from the use of the above infusion, given in small and frequently repeated doses.

It stopped the vomiting entirely, lessened the frequency of the discharge from the bowels, and rendered them more healthy, while a good secretion from the kidneys was maintained. One of the worst cases I met with was a little child aged 18 months.—The gastric and intestinal irritation was severe, and the discharges continued bloody, muco-purulent, and frequent, until a dangerous degree of prostration ensued. After trying remedies by the mouth and rectum, until I was well nigh discouraged, I took a tumbler full of the above named infusion, added to it 10 grs. of Tannate of Quinine, a few drops of peppermint, and sugar enough to make it pleasant. This was given in doses of a dessert spoonful so frequently repeated, that it was nearly all taken in 24 hours; at the end of which time the stomach had become quiet, the secretion from the kidneys good, and the discharges from the bowels much less frequent and more fecal. By continuing the infusion the patient was enabled to retain in addition a few drops of the emulsion of turpentine and laudanum three or four times a day, and fully recovered. I had intended to add many other items on the treatment of Dysentery, but present space will not permit.

